# Preclinical and Clinical Research Models of Prostate Cancer: A Brief Overview

**DOI:** 10.3390/life12101607

**Published:** 2022-10-14

**Authors:** Debasish Basak, Lisney Gregori, Fatema Johora, Subrata Deb

**Affiliations:** Department of Pharmaceutical Sciences, College of Pharmacy, Larkin University, Miami, FL 33169, USA

**Keywords:** prostate cancer, research models, preclinical, clinical, androgens, steroidogenesis

## Abstract

The incidence and mortality from prostate cancer (PCa) are on the rise which poses a major public health concern worldwide. In this narrative review, we have summarized the characteristics of major in vitro and in vivo PCa models including their utility in developing treatment strategies. Androgens, particularly, testosterone and dihydrotestosterone (DHT) activate the androgen receptor (AR) signaling pathway that facilitates the development and progression of castration resistant PCa. Several enzymes namely, CYP17A1, HSD17B, and SRD5A are essential to furnishing DHT from dehydroepiandrosterone in the classical pathway while DHT is formed from androstanediol in the backdoor pathway. The advancement in delineating the molecular heterogeneity of PCa has been possible through the development of several in vitro and in vivo research models. Generally, tissue culture models are advantageous to understand PCa biology and investigate the efficacy and toxicity of novel agents; nevertheless, animal models are indispensable to studying the PCa etiology and treatment since they can simulate the tumor microenvironment that plays a central role in initiation and progression of the disease. Moreover, the availability of several genetically engineered mouse models has made it possible to study the metastasis process. However, the conventional models are not devoid of limitations. For example, the lack of heterogeneity in tissue culture models and the variation of metastatic characteristics in xenograft models are obviously challenging. Additionally, due to the racial and ethnic disparities in PCa pathophysiology, a new model that can represent PCa encompassing different ethnicities is urgently needed. New models should continue to evolve to address the genetic and molecular complexities as well as to further elucidate the finer details of the steroidogenic pathway associated with PCa.

## 1. Introduction

**Figure 1:**
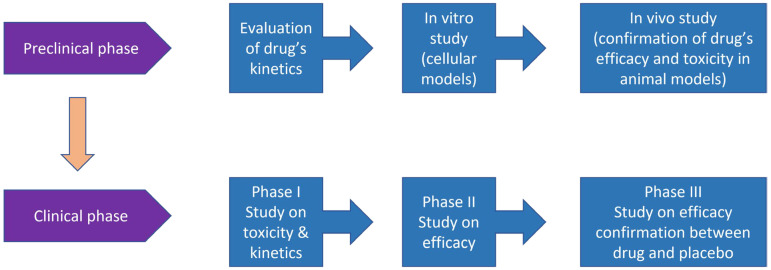
Schematic diagram of the flow of research from preclinical to clinical components.

Prostate cancer (PCa) is a frequently diagnosed malignancy that ranks second in cancer-related deaths in men in the United States [1]. The growth and survival of PCa have been attributed to the androgens that act by both stimulating proliferation and suppressing apoptosis. Thus, hormone therapy or androgen deprivation therapy has become the standard approach for PCa treatment. Despite an improved outcome with androgen deprivation therapy in the initial stages or in castration-sensitive PCa patients, the majority of the patients succumb to metastatic castration-resistant prostate cancer (CRPC). Androgen receptor (AR) amplification and mutations in AR are the most critical molecular aberrations, which basically trigger the development of CRPC. CRPC is characterized by the presence of androgen-refractory cells that are more aggressive and highly metastatic compared to androgen-dependent cells [2]. Although the recent FDA-approved agents (e.g., abiraterone, cabazitaxel) offer some benefist, the five-year survival rate of CRPC patients is only about 28.2% [3]. Figure 1 delineates the flow of research from preclinical to clinical components. Essentially, the development of PCa involves several steps that begin with the formation of prostate intraepithelial neoplasia (PIN). This arises due to the overexpression of glutathione S-transferase pi 1 gene (GSTP1), B-cell lymphoma 2 (BCL-2), and proto-oncogene MYC [4,5,6]. The subsequent loss of phosphatase and tensin homolog (PTEN), NK3 Homeobox 1 (NKX3.1), and retinoblastoma protein (RB1) results in prostate adenocarcinoma [7,8,9]. The condition is aggravated when metastatic PCa develops due to the mutations in AR, ATM, BRCA1/2, and loss of SMAD Family Member 4 (SMAD4) [10,11].

AR usually displays preferential binding to heat shock proteins (HSP) in its dormant form [12]. Upon binding of androgens to its ligand binding domain (LBD), AR gets activated and translocated to the nucleus. Subsequent binding of AR to androgen response elements (AREs) results in the transcription of several downstream genes. This also promotes the upregulation of various signaling pathways such as the PI3K/AKT pathway, which has enormous implications for different cancers [13]. Moreover, AR is closely linked to cell growth, proliferation, and metastasis. De novo steroidogenesis drives the maintenance of intratumoral androgens that may deliver an adaptive response in CRPC by activating the AR signaling pathway [14]. Androgens comprise different sex hormones of which testosterone and dihydrotestosterone (DHT) are the most prominent androgenic ligands of AR; others include androstenedione, androstenediol, and dehydroepiandrosterone. Usually, the Leydig cells in testes produce testosterone that gets converted to DHT by 5α-reductase (SRD5A) in the prostate [15]. Two types of steroidogenesis are observed that are designated as “classical” and “backdoor” pathways. In the classical pathway, pregnenolone and progesterone get converted to Dehydroepiandrosterone (DHEA) and androstenedione by the sequential hydroxylase and lyase activity of CYP17A1. After entering the prostate, these adrenal androgens yield testosterone with the help of enzymes HSD3B and HSD17B. Finally, testosterone gets converted to the more potent androgen dihydrotestosterone in the presence of SRD5A [16]. In the backdoor pathway, progesterone is first converted to pregnan-3,20-dione that ultimately gets converted to androstanediol via the enzymes CYP17A1 and HSD17B. Subsequent formation of DHT occurs from androstanediol by the actions of RDH5 and AKR1C [17]. PCa cells elicit an alteration in androgen sensitivity that is mediated by an overexpression of AR. This event is associated with the androgen independence of PCa cells. Overall, AR upregulation results in androgen-hypersensitivity that can lead to hormone-resistance in PCa patients [18]. Hence, AR antagonists are heavily employed in the treatment of PCa and a better understanding of AR function as well as AR sensitivity is of paramount importance to devise more effective PCa therapeutics. Apart from AR, a good number of markers are also available in PCa. These include but are not limited to cytokeratins, p53, PTEN, Rb, NKx3.1, Ki-67, β-catenin, EGFR, etc. 

Several PCa cell lines were developed in the early 1970s and 1980s, such as LNCaP, DU145, and PC-3 which are still indispensable in research [19,20,21]. Nevertheless, the common PCa cell lines are not devoid of limitations. For example, these cell lines do not accurately simulate the heterogeneity that is frequently observed in human tumors [22]. Therefore, patient-derived cancer models such as patient-derived xenografts (PDXs) are a better alternative to drug screening since they can more precisely simulate clinical responses in patients [23]. Presently, several PCa PDX models are used worldwide [24]. Human prostate tissue and PCa patients are one of the most sought PCa research models. In this narrative review article, we have summarized the major in vitro and in vivo PCa models that could be helpful for the investigators to decide on the appropriate models for conducting research and developing treatment strategies. Overall, PCa research models can be classified into preclinical and clinical models, where preclinical models are higher in both number and frequency of use. Preclinical models include primary and secondary cell lines, patient-derived or cell-line derived xenografts, transgenic mouse, knockout mouse, and cancer-related inflammation models. Likewise, clinical models are limited in nature and mainly comprised of human prostate tissues and patients. 

## 2. Cell Culture Models

### 2.1. Androgen-Dependent Cells: LNCaP 

The androgen responsive LNCaP cell line possesses the mRNA/protein expression of both AR and prostate specific antigen (PSA). This cell line was established from lymph node metastasis [19]. In the AR coding sequence, LNCaP displays a mutation in T877A that results in unsolicited affinity to different steroid compounds [25]. Their doubling time is 60–72 h and they express IGF-1-R, EGF/TGF-α-R, and FGF-R [26,27]. Moreover, they exhibit wild type (WT) p53 and PTEN inactivation [28,29,30]. Some sublines that were established from LNCaP showed the ability to grow in vivo after subcutaneous or orthotopic implantation. Among the sublines, LNCaP-LN3 produced lymph node metastases more frequently after being implanted into the prostate. Additionally, after intrasplenic implantation LNCaP-LN3 resulted in liver metastases. LNCaP-LN3 furnished high levels of PSA and showed less androgen sensitivity [31,32]. This cell line shows a high expression of SRD5A1, a very low level of SRD5A2, and no expression of CYP17A1 [33,34].

### 2.2. Androgen-Independent Cells

#### 2.2.1. PC-3

PC-3 cells that do not exhibit AR or PSA mRNA/protein were obtained from vertebral metastasis of prostate tumor. Their doubling time is about 33 h. This cell line expresses aberrant p53 with a nonsense codon that demonstrates a loss of heterozygosity [35,36]. They also express transferrin receptor [37,38] and their independent growth is due to elevated expression of TGF-α and EGF-R [39]. Due to its robust tumorigenicity, PC-3 remains a frequently used cell line that can grow easily in vivo [40]. It is worth mentioning that PC-3 is more characteristic of neuroendocrine rather than adenocarcinoma [41]. A metastatic variant of PC-3 is PC-3M. Tumors were excised from the prostate or lymph nodes and later, these tumor cells were reintroduced into the prostate. The cycle was repeated several times that generated the sublines, PC-3M-Pro4 and PC-3M-LN4. PC-3M-LN4 cells exhibited a greater level of lymph node, lung, and bone metastasis [32]. PC-3 shows very low expression of CYP17A1, moderate level of SRD5A1, and very low level of SRD5A2 [33,42].

#### 2.2.2. C4-2B

LNCaP C4-2B subline is androgen independent and has a doubling time of 48 h. These cells possess a greater metastatic potential than the parental LNCaP cell line. When these cells are injected intraosseous or intracardially into the immunodeficient mice, they produce osteoblastic or mixed osteolytic–osteoblastic lesions. Due to the elimination of different molecular properties of localized and treatment-naive PCa, this cell line attains the most undifferentiated state of PCa [40]. LNCaP and human osteosarcoma MS cells were subcutaneously co-injected into the mice from which C4 cells were generated. Later, C4 cells were subcutaneously injected into a castrated mouse followed by the culturing of the tumor cells and development of C4-2 cells. These cells finally resulted in a cell line that produced bone metastasis and this metastatic line was named C4-2B [43]. The steroidogenic enzyme expression in C4-2B is yet to be reported.

### 2.3. Wild-Type AR

#### 2.3.1. LAPC-4 

LAPC-4 cell line was generated from the lymph node metastasis of a hormone-refractory PCa patient through sequential subcutaneous xenografting into SCID mice. While developing this cell line, the explants from the PCa patients were xenografted into mice from which the tumor cells were later grown in culture dishes [44]. The doubling time of this cell line is about 72 h and the cells can grow subcutaneously, orthotopically, or intratibially in nude mice [45]. They express wild type AR, PSA, and PTEN and demonstrate p53 mutations (P72R and R175H) [46]. The available evidence also reported the expression of a basal epithelial and a luminal epithelial marker called CK5 and CK8 [44]. LAPC-4 displays the high expression of SRD5A1 [47].

#### 2.3.2. VCaP 

The VCaP cell line was generated from a hormone-refractory metastatic PCa patient [48]. This cell line displays AR, PSA, prostatic acid phosphatase (PAP), and retinoblastoma (Rb). Moreover, it expresses CK-8, CK-18, PTEN, and p53 which have an A248W mutation. Its doubling time is about 51 h and it grows reasonably well both in normal and castrated mice [49]. VCaP cells exhibit the TMPRSS2:ERG fusion gene that was reported to form tumors orthotopically [50]. It is also tumorigenic to SCID mice when injected subcutaneously [51]. VCaP cell line exhibits a high expression of CYP17A1 [52].

### 2.4. Normal Prostate Epithelium Cells RWPE-1 

The RWPE-1 cell line was obtained from a 54-year-old man’s prostate and human papillomavirus 18 (HPV-18) was used to make the cell immortalized [53]. Their doubling time is about 120 h and they express AR, PSA, and luminal prostatic epithelium markers, CK8 and CK18. They also display heterogenous nuclear staining for p53 and Rb proteins [54]. The epidermal growth factor (EGF) and fibroblast growth factor (FGF) promote the growth of RWPE-1 and TGF-β suppresses their growth [55,56]. It is important to note that the RWPE-2 cell line that can form a tumor in the nude mice after subcutaneous injection was developed by the transformation of RWPE-1 with Ki-ras [54]. The steroidogenic enzyme expression is absent in this cell line. Table 1 summarizes the primary characteristics of major PCa cell lines.

### 2.5. Drug Resistant Cell Lines

Like most other cancers, the development of drug resistance is frequently observed in PCa. Therefore, drug-resistant cell lines can be employed in research to identify novel molecular mechanisms of chemoresistance. Mohr et al. reported the generation of docetaxel-resistant PCa cell lines. In their study, they initially treated DU145 and 22Rv1 cells with 10 nM of docetaxel followed by an increase in drug concentrations (25 nM, 50 nM, 100 nM and 250 nM). They confirmed docetaxel resistance by colony formation assay and phenotypic characterization by qRT-PCR [57]. Hongo et al., generated cabazitaxel-resistant DU145 and PC-3 cells where they began cabazitaxel treatment at a concentration of 0.3 nmol/L and later, progressively increased the dose to a final concentration of 3 nmol/L. In this study, they found that DU145CR cells exhibited resistance against cabazitaxel-mediated G2/M arrest through the upregulation of ERK signaling and PC-3CR cells displayed amplified PI3K/AKT signaling [58]. In a follow-up study, they reported an elevated expression of AURKB and KIF20A in DU145CR compared to a non-CBZ-resistant cell line [59]. Kregel et al., reported the development of enzalutamide-resistant (EnzR) cell lines where they treated CWR-R1, LAPC4, LNCaP, and VCaP cell lines with 10 Μm enzalutamide for at least 6 months before carrying out experiments. They found that enzalutamide-resistant LNCaP and CWR-R1 cells presented enhanced castration-resistant and metastatic growth in vivo [60]. Liu et al., reported the development of abiraterone resistant C4-2B cells that were exposed to 5–20 Μm abiraterone acetate over 12 months. They demonstrated that the overexpression of AR-V7 was associated with resistance to abiraterone, which suggested that the addition of an AR-V7 inhibitor could be instrumental for treating advanced CRPC [61]. 

## 3. Xenograft Models

LNCaP cell line is very commonly used for developing mouse xenografts. This cell line was isolated from lymph node metastasis [19]. After isolation, its tumor forming ability in mice was about 50% through subcutaneous injection [62]. Nowadays, matrigel use can produce higher rates of tumor and form faster in male mice. It is unique due to its ability to express both AR and ER. They carry a mutated androgen receptor (AR) (T877A) [25]. Its tumorigenicity is inadequate in athymic nude mice and that is why LNCaP sublines were created that have better growth potential in vivo [31,32]. These include LNCaP-Pro3–5 and LNCaP-LN3–4. LNCaP-LN3 cells generate lymph nodes and liver metastases to a greater extent. 

The PC-3 cell line can also be used for developing mouse xenografts. Stephenson et al. reported the formation of lymph node metastases through orthotopic implantation of PC-3 cells [63]. PC-3M is a metastatic variant of PC-3. Basically, tumors were harvested from the prostate or lymph nodes, and these tumor cells were reinjected into the prostate. This resulted in the cell lines, PC-3M-Pro4 and PC-3M-LN4. PC-3M-LN4 cells were reported to display significantly more metastatic potential in lymph node, lung, and bone [32]. 

**Figure 2:**
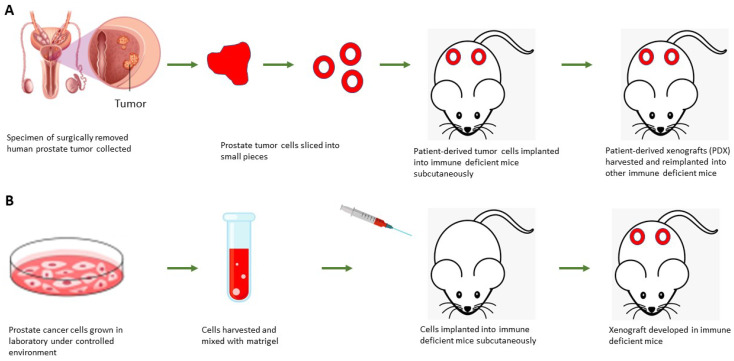
Illustrations of the development of (**A**) patient-derived and (**B**) cell-derived xenografts.

The DU145 cell line is another variant that is also used for developing xenografts. Both PC-3 and DU145 cells are androgen-independent and they do not express AR. Culig et al. created an LNCaP-abl cell line after culturing LNCaP cells 87 times in a medium devoid of androgen. These cells exhibit a greater amount of AR and display an amplified proliferation to androgen until passage 75. In nude mice, bicalutamide promoted the growth of LNCaP-abl xenografts while testosterone suppressed it [64]. LNCaP-IL6C cells have a tremendous growth potential in nude mice and this cell line was established after serial passaging in the absence of IL6 [65]. LNCaP-IL6C cells show an upregulation of cyclin-dependent kinase 2 and diminished expression of the tumor suppressors pRb and p27. Loberg et al., reported the generation of an androgen-independent PCa cell line through subcutaneous implantation and serial passaging of VCaP cells in castrated male SCID mice [66]. VCaP expresses high levels of PSA, prostatic acid phosphatase (PAP), cytokeratin-18, and wild-type AR [48]. Figure 2 describes the steps involved in cell and patient-derived xenografts development.

Although cell derived xenograft models are useful to assess the efficacy and mechanisms of drugs, they are devoid of heterogeneity and cannot mimic the complexity of the tumor microenvironment. Hence, PDX models are advantageous that are developed by relocating patient tumor samples into host animals. These models can better replicate tumor heterogeneity which is helpful to design more effective therapeutics. In 1976, Schroeder et al. first reported the transplantation of PCa tissues into nude mice [67]. Despite being a popular PDX model, nude mice can retain some humoral adaptive immunity and an innate immune system, thereby restricting PDX engraftment. SCID mice lack both T- and B-cells, but they are sensitive to radiation-induced DNA damage and can produce T and B cells with increasing age. IL2rg mutations resulted in the development of NSG, NOG, and NRG mice. NSG and NOG mice were reported to demonstrate an escalated sensitivity to drugs [2]. Considering the limitations of different models, orthotopic xenografting of tissue is accepted as the finest model that offers a more physiologically relevant microenvironment and the tumor take rate in this model is approximately 70% [68]. Table 2 illustrates the major features of PCa xenograft models.

## 4. Transgenic Mouse Models (Genetically Engineered Mouse, GEM)

### 4.1. PTEN

The deletion of phosphatase, PTEN takes place about in 86% CRPC [70]. While the homozygous PTEN knockout results in lethality to mouse embryos, the heterozygous PTEN^+/−^ knockout leads to multifarious lesions such as endometrial complex atypical hyperplasia, dysplastic intestinal polyps, lymphomas, and PIN [71,72]. This PTEN knockout model is useful to validate the role of the tumor microenvironment in the growth and progression of PCa. It is worth mentioning that Nkx3.1-PTEN double mutant mice can better mimic the early stages of human PCa since these mice have an increased tendency to develop high-grade prostatic intraepithelial neoplasia (HGPIN) [73]. 

Ding et al. generated PTEN^pc−/−^ Smad4^pc−/−^ mice that were later used to study the effects of combination therapy of a hypoxia-prodrug, TH-302, and checkpoint blocker. This combination therapy markedly extended the survival of PTEN^pc−/−^ Smad4^pc−/−^ mice [11]. Furthermore, Wang and colleagues reported that polymorphonuclear myeloid-derived suppressor cells (MDSCs) are a major source of infiltrating immune cells in PCa and their reduction can suppress the progression of PCa [74]. Liu et al., reported a novel PTEN knockout (*PTENadcre+*) mouse model where they generated prostate-specific Cre-LoxP genes through the delivery of adenovirus to the anterior prostate lobes. This model was a significant milestone to compare the age difference in PCa since the result showed that the old mice group has a greater frequency of PCa development and progression compared to young mice [75]. Table 3 depicts the genetic alterations and metastatic potential of genetically engineered PCa mouse models.

### 4.2. TRAMP

The transgenic adenocarcinoma of the mouse prostate (TRAMP) model was developed by Norman Greenberg and his group in 1995 [76]. In this model, the development periods of PIN and lymphatic metastases were 18 weeks and 28 weeks, respectively, and hence, it is a very useful model to study PCa pathology, prevention, and treatment [77,78]. This TRAMP model first presented castration-resistant disease progression [79]. In TRAMP mice, the rat prostate governs the expression of large and small SV40 T antigens through a prostate specific promoter probasin (PB or Rpb) [80]. Chloramphenicol acetyl transferase induced the expression of SV40 large T antigen and luciferase reporter assays confirmed the presence of two androgen receptor binding sites (ARBS) in the PB promoter [81]. The hemizygous TRAMP mice result in PCa with metastasis and can show different disease states such as mild intraepithelial hyperplasia and large multinodular malignant neoplasia. These mice can result in the formation of PIN and adenocarcinoma at the ages of 12 and 24 weeks, respectively in the dorsal and lateral lobes of the prostate. At the age of 12 weeks, the mice were castrated; however, this event did not impact the development of primary tumors or metastasis in most of the TRAMP mice. A major drawback of the TRAMP model is the development of neuroendocrine PCa that could be due to the loss of Rb and p53 [82,83]. A major drawback of the TRAMP model is the development of neuroendocrine PCa which could be due to the loss of Rb and p53 [82,83]. Therefore, the TRAMP model is more appropriate to study PCa of neuroendocrine origin. 

## 5. Knockout Models (Androgen Receptor Knockout (ARKO) Mice)

Androgen receptor knockout (ARKO) models are outstanding models since they are useful to study AR physiological roles in selective cell types within reproductive systems. To develop the ARKO mice, C57-B6/129/SvEv loxP-floxed AR mice were first generated and then they were mated with Cre recombinase expressing mice [84,85]. Floxed AR mice were mated with the transgenic mice that harbored Cre expression driven by the strong β-actin (ACTB) promoter human cytomegalovirus (CMV) promoter, or phosphoglycerate kinase (PGK) promoter [84,85,86]. Some other ARKO models are prostate epithelial ARKO (PEARKO) mice, Leydig cell–specific ARKO (L-AR−/y) mice, and germ cell–specific ARKO (G-AR−/y) mice [87,88,89]. AR signaling is important due to the fact that the global female ARKO mice exhibited impaired mammary gland development along with subfertility [90]. The ARKO male mice displayed reduced levels of testosterone and cancellous bone volumes than their wild-type counterparts. Moreover, they had a female-like appearance and ARKO female mice presented fertility impairment. The floxed AR mice are beneficial since they could be used to generate tissue-specific ARKO such as liver, breast, and prostate where the roles of AR could be determined [85]. Niu et al. (2008) reported that AR exerted distinct attributes in the stromal and epithelial cells of the prostate as evidenced in two models namely, inducible (ind)-AR knockout (ARKO)-TRAMP and prostate epithelial-specific ARKO TRAMP (pes-ARKO-TRAMP) models. They found that although AR was missing in both epithelium and stroma, pes-ARKO-TRAMP mice produced bigger tumors with a higher proliferation index [91]. Overall, the generation of ARKO model is of paramount importance since this offers a fresh avenue to determine the impact of androgens in the target tissues.

## 6. PCa Inflammation Models

The PCa inflammation models are heavily impacted by carcinogens, animal genetics, hormonal imbalance, fat-enriched diet, cholesterol, and advanced age. For example, an elevated expression of estrogen receptor-α (ER-α) and a decreased expression of ER-β and androgen receptor (AR) promoted the oncogenic effect of 17β-estradiol on NRP-152 cells [92]. Chronic inflammation was evident in 48 weeks old FVB/N mice due to the upregulated expression of estrogens such as 17β-estradiol as well as inflammatory markers namely, macrophages, neutrophils, and T-lymphocytes that resulted in prostatic intraepithelial neoplasias (PINs) [93]. Yatkin et al., showed that androgen replacement therapy was able to prevent the 17β-estradiol-induced inflammatory reaction in the prostates of castrated Noble rats [94]. 

The causative factors for prostatic infection are usually bacteria, fungi, uric acid crystals or urine reflux. All these stimuli result in the activation of proinflammatory cytokines in the prostate that promote tumor development. Reports suggest that an elevated expression of inflammasome complex in the prostate of a rat model may mimic an inflammatory state similar to BPH in humans [95]. Elkahwaji et al., demonstrated that inflammation triggers prostate carcinogenesis by inducing oxidative stress-mediated DNA damage and injury [96]. A high grade PIN developed in humanized mice due to prostate inflammation was the result of treatment with the carcinogen 2-amino-1-methyl-6-phenylimidazo [4,5-b]pyridine (PhIP). This was associated with an expression of AR while the expression of PTEN and P63 was diminished. When these mice were fed high-fat diet, they developed carcinoma in situ [97]. Overall, there lies a strong relation between persistent prostatic inflammation and PCa development.

## 7. Clinical Research Models of PCa

Clinical models of PCa research are primarily limited to human prostate tissues and PCa patients. Though preclinical models offer the advantage of ready availability and flexibility of treatment, clinical models provide the targeted endpoint of the research in question. 

### 7.1. Human Prostate Tissues 

Prostate tissues from healthy humans or patients represent a unique but challenging opportunity to understand the disease pathogenesis and key players that are involved in the disease. Higher expression of steroidogenic enzymes in human prostate homogenates (HPH) compared to cell lines offers significant advantages in terms of experimental ease and clinical relevance. Typically, human prostate tissues are homogenized which are termed HPH in laboratory terminology. Based on the maturity pattern, histology, and activity, the human prostate gland can be classified into several zones such as fibromuscular, transition, central, and peripheral [98,99]. Clinical studies have deduced that tumors from the peripheral zone have unfavorable long-term survival compared to tumors from the transition zone [100]. Biopsy and prostatectomy provide tissue samples for research purposes which are eventually categorized as normal or cancerous based on Gleason score and staging by a pathologist [101]. Both types of tissues are useful to understand normal prostatic physiology and pathological conditions that drive cancerous growth. Often, PCa patients are treated with hormonal agents (e.g., LHRH agonists) to shrink the size (neoadjuvant therapy) before radical prostatectomy [102]. These tissues are invaluable as both the biomarkers driving the disease as well as the effect of neoadjuvant therapy on disease biomarkers can be determined using these types of tissues. 

**Figure 3:**
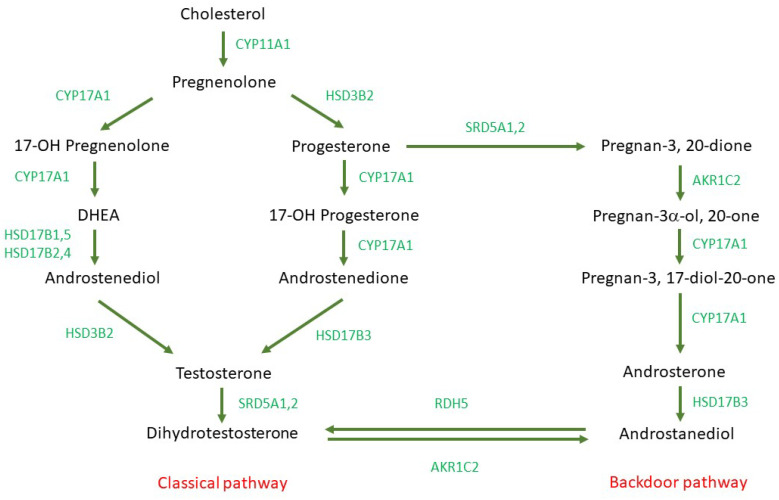
Human steroidogenesis pathway [34,103,105,106,107,110,111].

De novo steroidogenesis in the tissues reveals the intratumoral dynamics in CRPC [34,103]. Determination of protein expression by Western blot and immunohistochemistry is achievable [104]; however, gene expression profiling is not possible due to the flash frozen nature of the samples in wax blocks. HPH is one of the most reliable models to evaluate steroidogenic enzyme inhibition by experimental therapeutic agents [105,106]. Because steroidogenic enzyme levels are relatively higher in tissues than in secondary cell lines, the measurement of steroidogenesis is much more robust and measurable in HPH [34]. Both the classical and backdoor steroidogenesis pathways are observed in HPH with DHT as the end product [107,108,109]. SRD5A isoforms and CYP17A1 enzymes are detected in Western blot and activity studies in HPH [104] which provides mechanistic insight into steroidogenesis and its inhibitors. Figure 3 shows the steroidogenesis in humans including both classical and backdoor pathways.

### 7.2. Prostate Cancer Patients 

It is undebatable that PCa patients in clinical studies are the gold standard of research in this area. The species differences in metabolic landscape and tumor microenvironment between rodents and humans can pose a discrepancy in outcome [112]. While it is recognizable that humans should not be involved in research on PCa, or as a matter a fact for any disease, until the safety and efficacy of the compounds have been established with reasonable confidence, the pharmacokinetics, DDI, and other drug deposition-related studies face lesser regulatory stringency than during drug development stage. Identification of resistant treatment regimen is primarily derivative of post-approval clinical studies where lower treatment efficacy provide the impetus for research at the preclinical level to develop drug-resistant cell lines and research design [113,114]. 

## 8. Utility of Prostate Cancer Models in Drug Development 

Till today, a perfect model for PCa does not exist and this makes the research somehow ineffectual on a lot of occasions. Since there are legitimate ethical issues in relation to research on humans, the use of a mouse is still the choice of model. It is, indeed, imperative to find out the underlying cellular and molecular mechanisms of PCa development, progression, metastasis, and drug resistance to devise more effective therapeutic strategies. Despite being powerful, GEMMs are barely used to determine the effects of drugs. This is primarily due to their lengthy time course [115]. Similar to GEMMs, PDX is also used less frequently for drug studies [116]. Another reason is that metastatic lesions are uncommon in GEMMs. Extrapolatory evidence shows the absence of metastases in most of the cases where the mice have to be euthanized due to excessive tumor burden or sometimes due to immunosurveillance mechanisms [117]. The xenograft models that are developed by injecting human cell lines are most commonly used to investigate the effects of drugs. This is accomplished by subcutaneously injecting PCa cells, allowing the tumor to be palpable, treating it with drugs, and then determining the difference in tumor volume as per the study protocol. Some subcutaneous xenograft models can be beneficial to study metastasis and a drug’s effect based on the cell lines involved [118]. Orthotopic injection models that offer more patient-relevant tumor microenvironments are developed by seeding PCa cells directly into the murine prostate [119]. Nevertheless, this model is not devoid of disadvantages. For example, the establishment of the orthotopic model requires delicate surgical skills. Moreover, the measurement of tumor volume is complicated since the tumor in this model is not always visible. PDX models are useful as they have the advantages of cellular heterogeneity, molecular diversity, and histology of primary patient tumors. One disadvantage of this model is that reproducibility is comparatively low because of the heterogeneity of PCa [24]. Clinical models such as human prostate tissues and patients are obviously most desirable research models of PCa as the results are either directly or most closely translatable in understanding the disease, identifying drug targets, and/or developing treatment strategies. 

The use of PCa cells in cancer stem cell (CSC) research has also been reported that introduced a new horizon in PCa research. PCa stem cells were identified by Collins et al. from primary human PCa [120]. These CSCs can self-renew, differentiate, and result in tumor development. While currently available agents are effective against proliferating cells, CSCs are quite invulnerable that can render metastasis and drug resistance [121]. A very recent study by Li et al. demonstrated the use of PCa cells in CSC research where they showed that angiogenin and plexin-B2 regulate the stemness of prostate CSCs. In this study, they employed the CSCs that were cloned from PC-3, DU145, and LNCaP cells [122]. Similarly, Miolo et al. demonstrated that photoactivated 4,6,4′-trimethylangelicin (TMA) can reduce the expression CD44 when they conducted the study using DU145 cells [123]. Expression of CD44 serves as a viable marker of CSC and overexpression of CD44 is linked to PCa staging and response to therapy [124,125]. Silencing of CD44 results in inhibition of nuclear translocation of β-catenin in PCa [126]. Moreover, Assoun et al. characterized iPS87 PCa cell line and reported its stem cell-like properties [127]. These studies show that PCa cells possess enormous potential to be used in the development of novel treatment strategies involving CSC.

## 9. Limitations of Prostate Cancer Models and Future Direction 

The currently available PCa cell line models have several limitations. For example, these cell lines are devoid of heterogeneity that is frequently detected in patient-derived tumor samples [22]. Indeed, growing these cells in monolayer cultures is the reason behind this lack of heterogeneity. Moreover, these cell lines are grown under in vitro culture conditions that do not properly represent the human tumor microenvironment [128]. 

Compared to cell line models, patient-derived xenografts (PDXs) are preferred since they are grown in immunocompromised mice that can more precisely mimic the heterogeneity of human PCa cells. Additionally, they have their endocrine system unaffected, and they can be used in metastatic conditions. In the earlier days, the success with PDX was very limited; however, presently a good number of PDX models are being employed globally for evaluating the effects of different chemotherapeutics with enhanced success [129]. However, PDX models also entail some limitations. First, time constraint is a major drawback in these models since PDX engraftment and drug testing may take several months. Second, these models are beneficial for testing the effects of few drugs only and are not a good candidate for high-throughput screening. Third, metastatic characteristics of tumor cells may be variable due to the variation in clinical conditions. In addition, human prostate tissue models are difficult to obtain with access in research centers attached to a major PCa hospital. Furthermore, the swiftness of freezing the samples and method of freezing the samples after radical prostatectomy can have implications on the stability of proteins in those samples. Availability of pathologists to carry out the staging may restrict the proper interpretation of the PCa tissues and related biomarkers. 

Considering all these downsides, three-dimensional (3-D) cultures have emerged as a novel and suitable alternative to patient-derived cancer models. For example, spheroid culture model is created by using culture plates with low attachment properties and they offer substantial success rates [130]. Another model namely, 3-D organoid culture models can better imitate the morphological and genetic characteristics of actual tumors by virtue of retaining tumor heterogeneity [131].

## 10. Conclusions

The availability of a number of models has remarkably advanced the field of PCa research. Although the in vitro models offer convenience to study cancer-related pathways, in vivo models are useful to better understand the different steps of tumor progression. Nevertheless, the cell line models have a significant drawback of not producing the precise human tumor microenvironment and that is why in vivo models are of paramount importance since PDX in mice can better mimic the human tumor environment. Moreover, GEMs provide with a huge advantage of developing different types of PCa tumors. However, a model that can represent PCa of multifarious ethnicity is still lacking and hence, further investigation is unquestionably necessary to better understand the oncological drivers of PCa as well as to devise more effective therapeutic strategies. 

## Figures and Tables

**Table 1:** General properties of prostate cancer cell lines. Compiled from published literature [19,21,26,33,34,42,43,44,47,48,52,54].

Cell Line	Source	Doubling Time	PSA	AR	Markers	Steroidogenesis Markers	Advantage	Disadvantage
LNCaP	Lymph node metastasis	60–72 h	+	+	p53, absence of PTEN, vimentin	SRD5A1—high SRD5A2—very low	Sublines have growth potential in vivo	Insensitivity to androgen and mutated AR
PC-3	Vertebral metastasis	33 h	-	-	Absence of p53, loss of PTEN, TGF-α, EGFR	CYP17A1—very low, SRD5A1—moderate, SRD5A2—very low	Greater extent of metastasis	AR-negative
C4-2B	Xenograft of LNCaP cell in nude mice	48 h	+	+	p53, absence of PTEN	Undocumented	Very good growth potential both in intact and castrated mice	
LAPC-4	Lymph node of androgen insensitive patient	72 h	+	+	Mutation in p53	SRD5A1—high	Hormone-responsive	Possibility of androgen-independence if grown in female or castrated male mice
VCaP	Vertebral metastasis	51 h	+	+	Mutation in p53, PTEN, Rb	CYP17A1—high	Sensitivity to androgen; expresses PSA, and AR	The availability of wild-type TMPRSS2 and ERG genes obstructs the probe of TMPRSS2-ERG rearrangement in vitro
RWPE-1	HPE cells from the peripheral zone	120 h	+	+	P53, Rb	No expression	Beneficial to investigate the molecular mechanisms of benign prostatic epithelial cell proliferation	No tumor formation in mice

**Table 2:** Properties of xenograft models of prostate cancer. Compiled from published literature [31,63,66,69].

Cell Line	Source	Androgen Dependence	PSA	AR	Metastasis to Organs
LNCaP	Lymph node	+	+	+	Bone
PC-3	Bone	-	-	-	Bone
DU145	Brain	-	-	-	No
VCaP	Spinal cord	+	+	+	Bone

**Table 3:** Mouse model of prostate cancer developed by genetic manipulation. Compiled from published literature [76,70].

Model	Genetic Alteration	Metastasis to Organs	Time to Develop
PTEN	Cre recombinase-mediated removal of PTEN coding sequence between two loxP sites	Lymph node and lung	PIN: 6 weeks Invasive carcinoma: 9–29 weeks
TRAMP	Combination of prostate specific promoter PB and APR2 to induce SV40 large/small T antigen	Bone, lung, lymph node, adrenal gland, kidney	Mild hyperplasia: 8–12 weeks Metastatic lesion: 18 weeks

## Data Availability

Not applicable.
